# Anticipating Knowledge to Inform Species Management: Predicting Spatially Explicit Habitat Suitability of a Colonial Vulture Spreading Its Range

**DOI:** 10.1371/journal.pone.0012374

**Published:** 2010-08-25

**Authors:** Patricia Mateo-Tomás, Pedro P. Olea

**Affiliations:** School of Biology, IE University, Segovia, Spain; Smithsonian's National Zoological Park, United States of America

## Abstract

**Background:**

The knowledge of both potential distribution and habitat suitability is fundamental in spreading species to inform in advance management and conservation planning. After a severe decline in the past decades, the griffon vulture (*Gyps fulvus*) is now spreading its breeding range towards the northwest in Spain and Europe. Because of its key ecological function, anticipated spatial knowledge is required to inform appropriately both vulture and ecosystem management.

**Methodology/Findings:**

Here we used maximum entropy (Maxent) models to determine the habitat suitability of potential and current breeding distribution of the griffon vulture using presence-only data (N = 124 colonies) in north-western Spain. The most relevant ecological factors shaping this habitat suitability were also identified. The resulting model had a high predictive performance and was able to predict species' historical distribution. 7.5% (∼1,850 km^2^) of the study area resulted to be suitable breeding habitat, most of which (∼70%) is already occupied by the species. Cliff availability and livestock density, especially of sheep and goats, around 10 km of the colonies were the fundamental factors determining breeding habitat suitability for this species.

**Conclusions/Significance:**

Griffon vultures could still spread 50–60 km towards the west, increasing their breeding range in 1,782 km^2^. According to our results, 7.22% of the area suitable for griffon vulture will be affected by wind farms, so our results could help to better plan wind farm locations. The approach here developed could be useful to inform management of reintroductions and recovery programmes currently being implemented for both the griffon vulture and other threatened vulture species.

## Introduction

In the management and conservation of species it is fundamental to determine their current and potential distribution, as well as both the amount and arrangement of suitable habitats in a landscape [1], [2]. In this context, predictive modelling of species' distribution has become a fundamental tool [3], [4], as it enables the quantification of relationships between a species and its environment, and making spatially explicit decisions about conservation planning [4], [5]. Presence/absence models are frequently used to predict species' distribution, but there is a common problem related to the uncertainty in determining absences [6], especially where the species does not occupy all available suitable habitats [7]. This is frequently the case in invasive [1] or native species whose distribution ranges have been either reduced or are still spreading. In such cases, methods to model presence-only data such as maximum entropy modelling (Maxent) [6] become powerful tools in predicting species' potential distributions across new areas [1], [8].

The expansion of a species into a new area can lead to conflicts or new local conservation problems, although in other cases it can also provide benefits to the ecosystem [9], [10]. Therefore, the anticipated spatial knowledge of these possible outcomes can be a very useful piece of information in order to advance the planning needed in management and conservation.

Vultures are among the most threatened avian guilds of the world [11], with 11 out of 21 species threatened [12]. This conservation status is partly due to the fact that vulture populations have developed a strong reliance on ever-changing human activities (e.g. livestock, feeding stations, hunting; [13] and references therein) [14], [15]. Since they are the only vertebrate obligate scavengers, the important ecosystem services provided by vultures (e.g. recycling of nutrients, limiting spread of diseases) are difficult to replace, and thus scavenging is the most threatened service worldwide [11], [16]. The importance of this vulture ecosystem service has recently been noted in Europe, where sanitary restrictions derived from the bovine spongiform encephalopathy (BSE) have produced such a high impact on scavengers and ecosystems that the European Union has been forced to modify the legislation (i.e. Regulation (EC) No 1069/2009) [13]. The griffon vulture (*Gyps fulvus* Hablizl) is one of the species most affected by these legislation-driven management changes [17], [18].

The griffon vulture is a colonial cliff-nesting scavenging raptor widely distributed from the Mediterranean countries to India, occupying also areas in the north of Africa [19]. In Europe the species, formerly common [20], experienced a strong decrease during the 1950's and 1960's, and became almost extinct in many countries (e.g. France) [21]. The implementation of protection laws prohibiting hunting and the commercialisation of vultures, and the ban of poison use during the 1970's and 1980's, enabled the recuperation of the species in some countries (i.e. Spain, Portugal, former Yugoslavia) [22]–[24]. However, despite the species being classified as of Least Concern by the IUCN [18], it is locally threatened in some regions where recovery programmes are carried out (e.g. Italy, the Balkan countries; A. Camiña, pers. comm.) [25].

In Spain, its main European stronghold, the griffon vulture population has strongly increased during the last three decades [26], but its distribution range has almost not increased [27]. Nonetheless, in the north-westernmost edge of the species distribution in Spain and Europe, the griffon vulture has sharply increased both its distribution range (by 1068%) and population during the last three decades (authors unpubl. data) [26]. In the north of this area, there are references of griffon vulture breeding colonies as early as 1930 [23]. However, in the 1950's and 1960's the species sharply declined, disappearing from most of the area [23]. In the 1980's, the griffon vulture populations recovered and the species started its expansion across the area, colonizing new places where it was never registered as a breeder before [27], [28]. Today, the population is expected to continue increasing and to colonize new areas (authors, unpubl. data).

In this context, identifying the new potential areas to be colonized by the griffon vulture is a highly useful tool for wildlife managers in order to identify target areas for monitoring and management of the species and the ecosystems where the species is present [16], [29], [30]. For example, spatially explicit predictions of habitat suitability for the vulture can be compared with the spatial distribution of potential threats [4] such us wind farms or potential competition with other species for breeding places (e.g. the endangered egyptian vulture, *Neophron percnopterus* Linnaeus) or for food resources (e.g. brown bear, *Ursus arctos* Linnaeus; other scavenger bird species; see below) [10], [18]. The benefits provided by the species may also be spatially extrapolated, such as the ecosystem services exerted by vultures through the elimination of carcasses [16].

The aims of this study are: i) to identify the environmental variables defining suitable habitat for the griffon vulture using presence-only data, ii) to identify areas that have suitable habitat (and thus a high probability) for future colonization at the northwestern edge of the species' distribution in Spain and Europe and iii) to generate a spatially explicit predictive map of habitat suitability to inform conservation and management planning of both species and ecosystems in the region. We perform these analyses accounting for two methodological topics often overlooked: multi-metric model evaluation and spatial autocorrelation.

## Results

**Figure 1:**
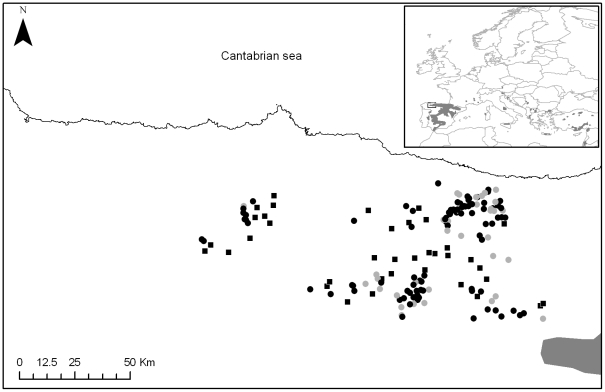
Study area. The study area (black rectangle in the inset) is located at the northwestern edge of the distribution of the griffon vulture in Spain and Europe (in dark grey in the inset). Black dots in the main map correspond to breeding colonies of the training data set (*N* = 87). Grey dots are breeding colonies using in the test data set (*N = *37). Black squares are available cliffs used as pseudo-absence data (*N* = 37).

The number of griffon vulture breeding colonies (i.e. presences) amounted to 124. Accordingly, the Maxent model was performed using 87 training and 37 testing presences (Fig.1). This sample size was sufficient for use in Maxent modelling methods [6].

### Environmental factors

**Figure 2:**
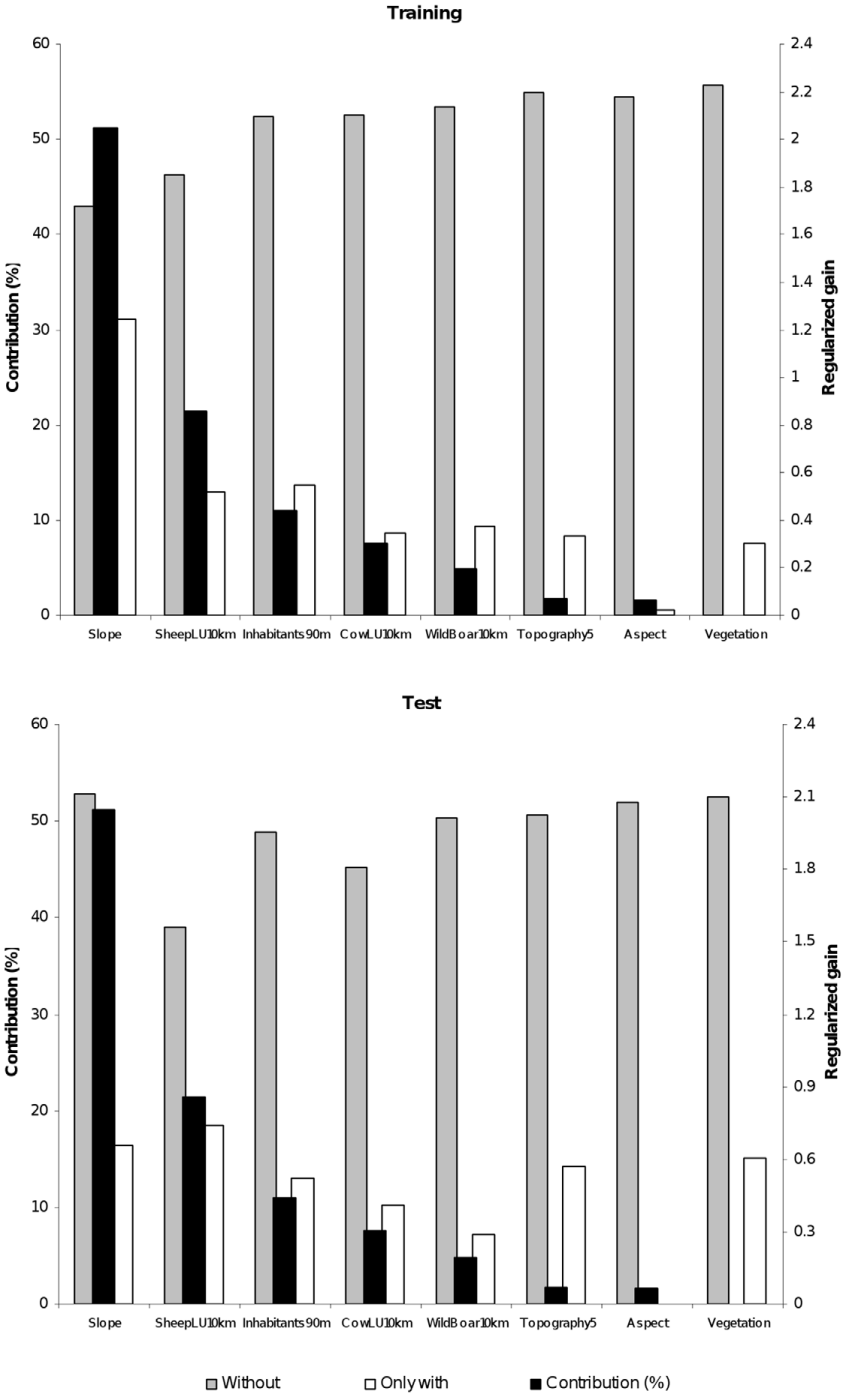
Importance of environmental variables according to Maxent models. Importance of environmental variables according to the training (above) and the test (below) Maxent models. Black bars correspond to the per cent contribution of each variable to the model (left axis). Grey bars represents the jackknife results of models without that variable and white bars the jackknife results of models with only that variable (right axis).

**Figure 3:**
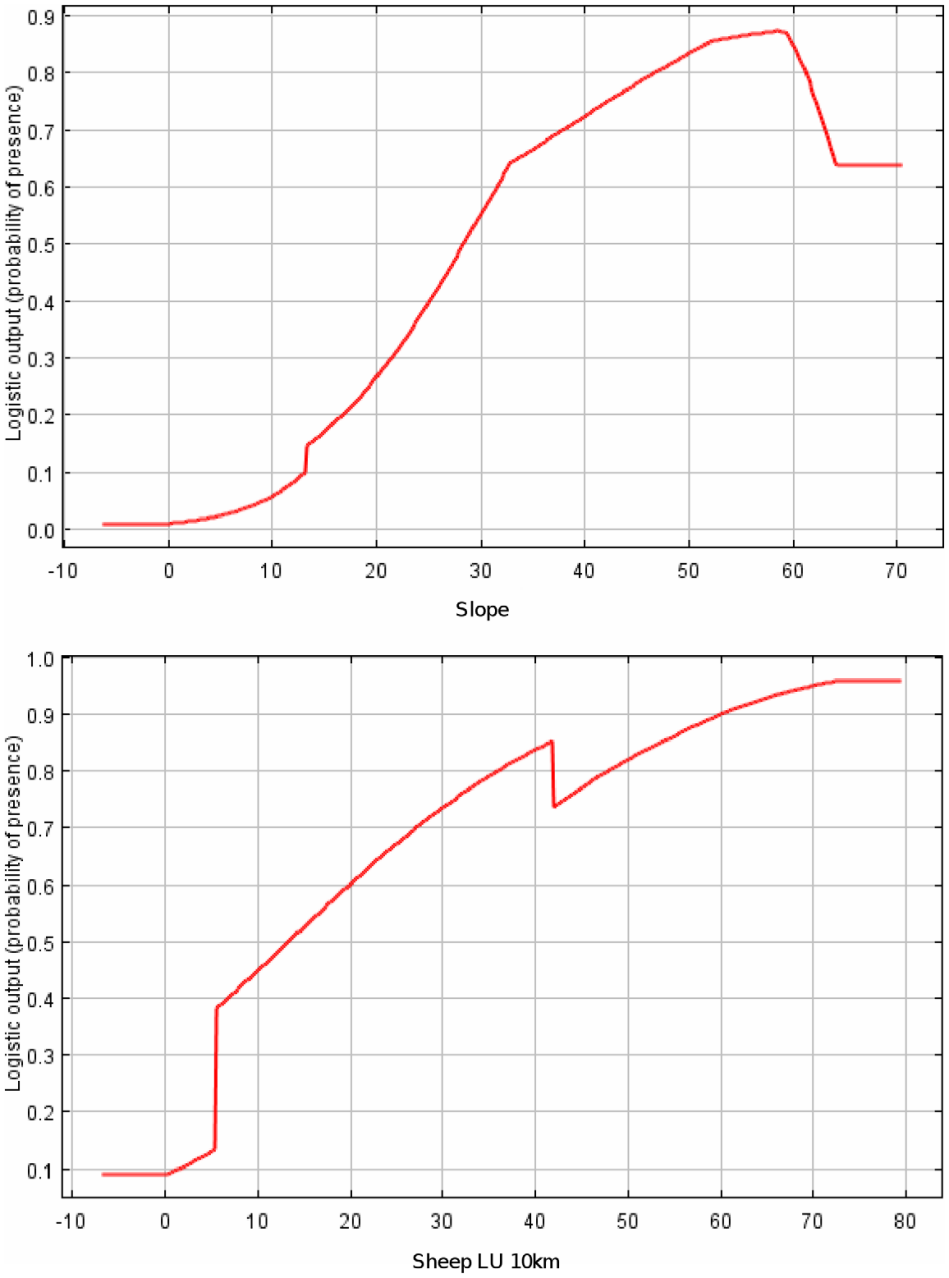
Response curves for the most significant predictors of griffon vulture habitat suitability according to the MAXENT model.

According to Maxent jackknife analysis, the most important environmental variables in determining habitat suitability for griffon vulture were slope (51.24% of contribution) and sheep and goat LU density within 10 km (21.45% of contribution; Fig. 2 and 3). Both variables had the highest gain when used alone in both training and test models respectively (Fig. 2). Accordingly, the areas most suitable for griffon vulture were those with higher topographic irregularity and an abundance of sheep and goats (Fig. 3). Other food resources such as hunting activity of wild boar and cow LU within 10 km radius around the colonies also increased habitat suitability for griffon vulture. The species seems to avoid highly human populated areas with dense vegetation cover (Fig. 2). Although aspect was included in the model, this variable provided almost no information (Fig. 2).

The overall gain in the training model decreased the most when slope was withdrawn from calculations, indicating that this variable provides a substantial amount of useful information that is not already contained in the other variables (Fig. 2). Similarly, sheep and goat LU was the next variable, providing the most useful information to the training model. In the test model the higher decrease of the overall gain occurred when removing sheep and goat LU (Fig. 2), indicating that this variable generalizes better, allowing a higher model transferability [6].

### Model predictions

**Figure 4:**
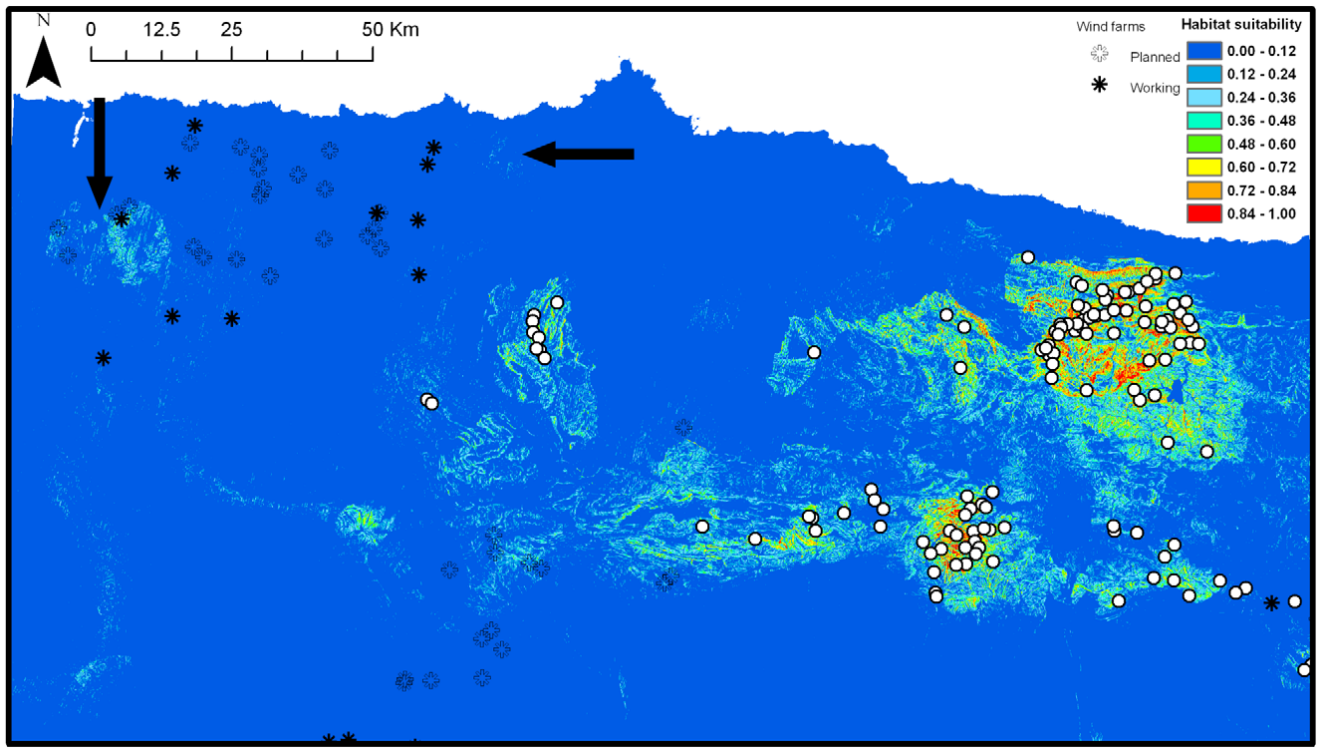
Habitat-suitability map for griffon vulture in the Cantabrian Mountains. White dots are breeding colonies. Black dots are griffon vulture roosts. Black arrows point to areas with historical records on griffon vulture colonies (i.e. 1950's: vertical arrow, 1930's: horizontal arrow). Both currently working and planned wind farms within the study area are also showed.

Considering a threshold of 0.24 (i.e. 10^th^ percentile presence value and maximised sum of sensitivity and specificity), around 7.5% (∼1,850 km^2^) of the study area would be suitable breeding habitat for the griffon vulture. Most of this suitable area (∼70%) is already occupied by the species, but, according to our model, the griffon vulture could expand its breeding range in the Cantabrian Mountains up to 50–60 km towards the west (Fig. 4). The current species range could increase up to 1,782 km^2^ if griffon vulture occupies all the areas predicted as suitable by our model (calculated by Minimum Convex Polygon, MCP) [27]. According to model predictions, the habitat suitability of the still available areas was significantly lower than that currently occupied by the species (i.e. mean±SD: 0.44±0.08 vs. 0.64±0.16, respectively; Wilcoxon test: W: 651, *P*<0.001).

The habitat suitability of the areas included within a 10 km radius around the currently working wind farms averaged 0.02 (SD: 0.02). This value increased to 0.03 (SD: 0.03) when the planned wind farms were also considered (Fig. 4). Only 0.01% of the area within 10 km around the currently working wind farms had a habitat suitability ≥0.24 (i.e. the threshold selected in our model to classify suitable habitat for the griffon vulture, see above). This value greatly increased up to 2.22% when considering both currently working and planned wind farms. The currently working wind farms only affect 0.02% of the total area identified by our model as suitable habitat (i.e. habitat suitability ≥0.24), but this value increases up to 7.22% when considering both the current and planned wind farms (Fig. 4).

The validation test of the Maxent package provided very high estimates for both training (0.976) and test (0.949) AUC (Table 1). Although higher, these results were congruent with those of our independent evaluation (Table 1), highlighting a very good model performance. Of the 37 breeding colonies of the test data set, the model correctly classified 31 (83.78%), the same percentage as the correctly classified absences (i.e. 31 out of 37).

**Table 1:** Evaluation of model performance.

		Metric	Value
		Training AUC	0.976
Prensence-only data	MAXENT		
		Test AUC	0.949
	Threshold-	AUC	0.899*
	independent	COR	0.657*
Presence/absence data		True skill statistic (TSS)	0.676
	Threshold-	Correct classification rate	0.838
	dependent	Sensitivity	0.838
		Specificity	0.838
		Cohen's Kappa	0.784

Statistical metrics used to evaluate model performance by MAXENT (i.e. presence-only data) and with presence/absence test data.

Training AUC, area under the curve for the training data set; Test AUC, area under the curve for the test data set; COR, Pearson correlation coefficient.

*P<0.05.

The model residuals did not show a significant spatial autocorrelation (Fig. S1).

## Discussion

Our results identify both the most suitable areas to be occupied by the griffon vulture and the factors determining their quality, enabling the anticipation of their monitoring and management. The results clearly show that the griffon vulture could expand its breeding range west of the Cantabrian Mountains, increasing the total species range to around 1,782 km^2^ (Fig. 1 and 4) According to our model, this expansion could mainly occur through the center-southern part of the area, where habitat suitability is higher (Fig. 4). In fact, up to eight vulture roosts (authors, unpubl. data) [15] have been reported in the southern corridor identified by our model as suitable habitat (Fig. 4). These roosting sites could be considered as clues about future breeding colonies, as several sites firstly used as roosts have become breeding colonies within the study area (F. Jubete, pers. comm.; authors, unpubl. data).

Suitable habitat for the griffon vulture was positively related with slope, a proxy of cliffs where the species breed. In fact, rocky surface also had a positive influence on habitat suitability in the study area (Fig. 2). The positive influence of livestock, specially sheep and goats, on habitat suitability seems to be related with the importance of extensive livestock as a fundamental food resource for this species [15]. In fact, livestock has been highlighted as an important variable to determine cliff occupancy by griffon vulture in other areas [31]. The negative influence of human density on habitat suitability could be related to increasing disturbances and/or to a low livestock rearing activity in highly human populated areas. Although the griffon vulture has a relatively high tolerance to human presence [31], [32], a negative effect of high human density has also been highlighted in other areas [33]. In fact, the bigger gap within the species distribution, located in the north of the study area, corresponds to the most highly human populated area (Fig. 4).

The model residuals did not show a significant spatial autocorrelation, highlighting the importance of the selected environmental factors in structuring the species distribution [34]. Nonetheless, although non-significant, the positive autocorrelation existing within 7 km (Fig. S1), could be explained by endogenous factors such as the conspecific attraction [35] which characterizes this species [21]. The good evaluation results of our Maxent model from both threshold-dependent and -independent tests (Table 1) highlight a high ability of this model to predict habitat suitability for the griffon vulture and thus potential areas to spread within the study area. However, despite the fact than one of the most important applications of habitat suitability models is to provide information on new areas of species occurrence [29], it is important to question if model predictions are accurate [34], [36] **.** In this context, several dispersal limitations such as geographical barriers or competition are not accounted for in most habitat suitability models [37]. In our case, griffon vulture dispersal ability is high enough to guarantee that it could colonize all the study area and no geographical barriers seems to exist which could limit this expansion. In fact, up to twelve vulture roosts (authors, unpubl. data) [15] have been reported within the suitable habitat predicted by the model, indicating the species' ability to access these areas (Fig. 2). In addition, there are several evidences of sites historically used by griffon vulture within the area highlighted as suitable by our model. Historical data refers to three extinct breeding colonies existing until 1954 in the northwesternmost edge of the study area [23], which is predicted by our model as suitable habitat (Fig. 4). Our model also highlights some suitable places for the griffon vulture in the center-northern part near the coast, where there is historical evidence of one breeding colony in 1930 (Fig. 4) [23]. The griffon vulture has no natural enemies which could limit its expansion, however, some human-related limitations such as illegal poison use and wind farms could offer resistance to the western expansion (see below).

### Management and conservation implications

Predictive models are a useful tool for wildlife managers to make better decisions about biodiversity management and conservation [29]. Our results identify suitable habitat for the griffon vulture, which is expanding within the Cantabrian Mountains. Accordingly, we provide valuable information about both i) possible areas to be colonized by the griffon vulture and ii) environmental factors determining habitat suitability for this species.

Our results could be useful in managing another aspect related to the impacts that the establishment of griffon vultures could have on the ecosystem. Griffon vulture scavenging services (see Introduction) [11] provide an economic method of carcass disposal [16]. This could be highly beneficial in the study area, where extensive grazing and hunting are important activities which generate a considerable amount of carcasses [14], [15]. The prediction of our model could help when planning carcass disposal systems within the study area (e.g. industrial disposal of carcasses, with transport and incineration *vs.* more natural systems based on scavenger consumption) [16]. This planning is also particularly important considering that the griffon vulture can influence carcass use (e.g. through reducing their availability) by other threatened scavengers in the study area such as the brown bear or the wolf (*Canis lupus* Linnaeus) [10], [38].

The griffon vulture has been reported occupying nests of other raptors, some of them highly endangered such as the bearded (*Gypaetus barbatus* Linnaeus) or Egyptian vultures [39] **.** The important population of Egyptian vulture in the study area has its main nuclei located within the south-central corridor predicted as potentially suitable area for the griffon vulture [40] **.** Since several cases of griffon vulture occupying Egyptian vulture nests have been reported in this study area (authors, unpubl. data), our predictive model allows for recognition of potential areas of possible conflict between both species.

Livestock, especially sheep and goats, is a relevant factor of the habitat quality for both griffon (this study) [15] and Egyptian vultures [41], [42] in the study area. The decrease of extensive rearing of sheep and goats is expected to negatively influence griffon vulture population, as previously stated for both this and other vulture species in the area [15], [42]. Our model can identify the target areas where extensive livestock should be promoted to benefit griffon vulture conservation.

The potential for expansion of the species towards the west, as predicted by our model, could generate a new conservation problem related to the increasing presence of wind farms in this part of the study area and especially in the south-central corridor [40], [43], where spatial overlap between suitable areas to be colonized by the griffon vulture and planned wind farms occurs (Fig. 3). Although according to our results, the suitable area potentially affected by wind farms is not high (7.2%), griffon vulture is the raptor most frequently killed by collision with wind farms [44]. In fact, in some areas of Spain, a huge griffon vulture mortality of up to 8 vultures/turbine/year has been reported [43]. Our model could help to better plan wind farm locations in order to avoid or reduce this emerging conservation problem.

Poison use was highlighted as one of the main causes of vulture decline in the study area in the past, resulting in the disappearance of up to three breeding colonies [23]. More recently, at least 59 griffon vultures have been poisoned in the last decade in the study area (local authorities, pers. comm.; Antídoto Program database, WWF/Adena). The hugely negative effects of poison on the species [45] could increase in the future since this illegal practice seems to be frequent in the western part of the study area (Antídoto Program database, WWF/Adena). Again our model, by identifying areas likely to be occupied by griffon vulture, would enable advanced methods of poison control therein.

If incorrectly accounted for, these three factors (i.e. livestock decrease, wind farms and illegal poison use) could have a key role in reducing habitat suitability for the species in the Cantabrian Mountains.

Our modelling approach was developed using the current values of the explanatory variables (Table 2). Future scenarios for some factors affecting vultures are rather uncertain [13]–[18]. Nonetheless, some derived effects of these changing factors are, however, expected to be somewhat predictable. For example, the decrease of some farming practices such as sheep and goat rearing could result in a decrease of suitable habitat not only by reducing food available but also by increasing vegetation coverage, which could make foraging more difficult for vultures. Simultaneously, the increase of vegetation coverage could favour wild ungulate populations. At any rate, in order to adequately extrapolate our model in the study area in the future, those variables identified as important by the models and with potential for changing -for example, through habitat management or climate change- should be conveniently updated.

**Table 2:** Environmental variables.

*Name*	*Description*	*Hypotheses*
HABITAT		
*Slope*	Slope angle of the terrain (degrees)	Griffon vulture is a cliff-nesting raptor, so areas with higher slope will have more cliffs and therefore higher habitat suitability
*Elevation*	Altitude of the terrain (m.a.s.l.)	Lower elevation can provide protection against inclement weather
*Aspect*	Terrain exposure classified into eigth categories: north (0°–22.5°; 337.5°–360°),northwest (292.5°–337.5°), west (247.5°–292.5°), southwest (202.5°–245.5°), south (157.5°–202.5°), southeast (112.5°–157.5°), east (67.5°–112.5°) and northeast (22.5°–67.5°)	S or E exposures can provide protection against inclement weather
*Topography3*	Difference between elevation of the cell and the mean of those included in a moving window of 3×3 side	Ruff terrain can mean more rocky cliffs for nesting but can also increase energy costs of movement
*Topography5*	Difference between elevation of the cell and the mean of those included in a moving window of 5×5 side	
*Coverage*	Surface coverage according to vulture accesibility: villages (0), forests (1), shrub (2), pasture (3) and rock (4)	Open areas facilitate carcass detection and access
*Inhabitants* *	Density of inhabitants	Human presence can increase disturbance to breeding colonies but can also provide predictable sources of food
FOOD		
*LU* *	Density of livestock units 1 cow = 5 livestock units; 1 sheep or goat = 1 livestock unit	Livestock is an important food resource for the species
*CowLU* *	Density of livestock units of cow	
*SheepLU* *	Density of livestock units of sheep and goat	
*RedDeer* *	Density of captures of red deer	Game species are an important food resource for the species
*WildBoar* *	Density of captures of wild boar	

Environmental variables used to model griffon vulture distribution in the study area.

*Variables calculated at three scales: 90 m-pixel, 3.5 km radius and 10 km radius, centred at the breeding colony.

We have used some methods which could simplify the extrapolation of our model to other areas (i.e. reachable background data and exploring predictions by using roosts and former locations) [36]. However, the problems associated to the use of species distribution models for extrapolation require new methods and techniques currently under development [36]. In the meantime, to effectively apply this modelling approach outside the study area, local presence data should be used together with updated explanatory variables. Importantly, correlation between variables should be tested, since it could change through both space and time [36]. Additional tools are being developed and implemented to assess the similarity between new environments and those of the training sample, providing ways to assess the applicability of our results to other areas [36]. According to our model, food availability (i.e. sheep and goat LU) will allow a high model transferability (see Results). However, due to the large variability of food resources available to scavengers in different regions of the world (i.e. feeding stations, extensive livestock, wild ungulates,...), variables describing food availability should be adapted to the local characteristics of the modelled area. Where the species is locally extinct, the input of the model could take advantage of data on the historic distribution of the species (Fig. 4).

According to the considerations above, the modelling approach here developed could be used to manage griffon vulture populations in other regions of the world. It could be especially useful to identify the habitat suitability of those areas with specific actions such as reintroductions or recovery programmes for both the griffon vulture (e.g. the Balkans, Israel, Italy; A. Camiña, pers. comm.) [25] and other vultures, such as critically endangered white-rumped (*Gyps bengalensis* Gmelin), the Indian (*Gyps indicus* Scopoli) and the slender-billed (*Gyps tenuirostris* Gray) vultures [46]. In fact, identifying the location of remaining colonies of these species in Asia is one of the conservation actions proposed, along with the monitoring of those areas where it is expected their populations may not have crashed (i.e. the white-rumped and slender-billed vulture in Cambodia and Myanmar and the Indian vulture in southern India) [47]–[49]. Accordingly, our modelling approach could be useful to identify both suitable habitats, which could provide information concerning where to look for new colonies of these species and where to release individuals from the several captive breeding programmes that are now working [47]–[49]. The application of this approach could also enable the identification of suitable areas where anticipation of some conservation measures is of huge importance for the conservation of these species. For example, it could help to identify both optimal locations for supplementary feeding stations and suitable areas in which to promote the exchanging of diclofenac with meloxican, which is currently restricted only at the proximity of the breeding colonies in some places (i.e. Nepal) [47]–[49].

## Materials and Methods

### Ethics statement

All research was conducted using existing databases and breeding places were surveyed from an appropriate distance to avoid disturbance of the birds. Permission for the research was granted when necessary by regional administrations (Junta de Castilla y León, Principado de Asturias and Gobierno de Cantabria) and Picos de Europa National Park.

### Study area

The study area covers approximately 24,639 km^2^ in NW Spain, corresponding mainly to the Cantabrian Mountains (Fig. 1). The region is recognized for its high biodiversity, holding several protected areas (i.e. >55% of the total area) such as ten biosphere reserves and 20 sites of communitarian interest [30]. The area is mainly located within the Temperate climatic region, although there is a transition area to the Mediterranean region in the south. The eastern part corresponds to the highest and widest area and is characterised by the presence of high, rocky mountains, up to 2,648 m.a.s.l. The western part combines high, rocky mountains with lower altitudes mainly dominated by forests.

### Species distribution

Data on the species distribution in the study area were obtained from national [26], [28], [50], [51]and regional censuses (FAPAS, F. Jubete, Picos de Europa National Park, pers. comm.) [27]. We validated this data through field work developed during the period 2005–2008. All these censuses followed a similar methodology [50], allowing us to minimize potential pitfalls derived from different sampling methods [6]. Moreover, the high sampling intensity developed over the entire area by many different agents (i.e. environmental authorities, researchers and volunteers), guarantees the absence of spatially autocorrelated sampling and associated problems [52].

In order to ground validate our model we used presence/absence data [53]. Presences were those breeding colonies not included in the Maxent training model (see below). A breeding colony was considered a cluster of breeding pairs located more than 1 km apart from each other [21] and located in cliffs with different physical characteristics such as rocky substrate or aspect. In other words, distances between nests within a cluster of breeders are smaller than distances between nests from different clusters.

We randomly selected the same number of available cliffs (i.e. pseudo-absences) as that of presences in the test data set. Available cliffs were selected within the current breeding range of the griffon vulture (Fig. 1). This minimised the bias due to the fact that available cliffs were outside the current breeding range of the species. Places used by griffon vultures have white droppings which are both highly visible and durable (authors, per. obs.). This allows us to reliably identify pseudo-absences during field surveys as available cliffs not used by the species (i.e. without white droppings).

### Environmental data

To model the species distribution we considered 11 variables related to habitat structure and food availability (Table 2). Climatic conditions such as precipitation or temperature were not used, as they are expected to have low explanatory power at local scales [54]. Moreover, these predictor variables, whose effect on species may vary across the study area, are not recommended when predicting potential distributions [37].

The habitat structure was defined according to topographic variables such as slope, aspect and elevation, derived from a 90-m resolution digital elevation model (DEM). This resolution was considered enough according to the mean length of the cliffs used by the species (mean±SE: 968.0±127.4 m; range: 62–3183 m). Additionally, we calculated two topographical position indices to define the position of the cell along the topographical gradient (valley, middle slope, ridge top) [55]. Vegetation cover was derived from the 3^rd^ National Forest Inventory [56] and reclassified according to accessibility for vultures (Table 2). Inhabitant density was derived from official data [57]. In order to quantify food availability, we considered the density of both cows and sheep and goats, whose influence on the species distribution has been previously stated [15]. The density of hunting episodes on red deer and wild boar was also considered as they have been highlighted as an important food resource for the species in the study area [14]. We considered three spatial extensions (i.e. 90 m-pixel, 3.5 and 10 km radius) according to cliff location and to the area most intensively used by vultures around roosts and colonies [14], [15], [51]. Data on livestock and hunting activity were obtained from official databases (Gobierno de Cantabria, Junta de Castilla y León, Principado de Asturias, Xunta de Galicia, pers. comm., see 14 and 15 for further details).

Illegal poison use was not included in the model despite having a high impact on the griffon vulture [45]. The huge difficulty in detecting all the poisoning events occurring in the study area prevented us from using a reliably spatial distribution of this variable. This decision was made to avoid introducing a high uncertainty in the model results [34]. On the other hand, the fact that most of the wind farms are in prospect (Fig. 4) [40], prevented us from including them in our model. Nonetheless, we assessed the possible conservation conflicts between wind farms and griffon vulture by comparing spatially explicit predictions of habitat suitability for the species with the spatial distribution of currently working and planned wind farms. We considered a radius of 10 km around the wind farms, as this is the area most intensively used by vultures around roosts and colonies [14], [15], [32].

### Model building

Environmental suitability was modelled using maximum entropy modelling (Maxent), a machine-learning process that uses presence-only data [6]. We selected a method only requiring presence data since these methods have been shown to outperform regression methods where a species does not occupy all available suitable habitats [7]. Maxent has been highlighted as the most effective method requiring presence-only data [37], [58]. Maxent models a probability distribution of habitat suitability over the study area. This probability is not of occurrence, but rather a value representing the relative suitability of the environmental constraints for the target species in each pixel in the study area [6].

We used version 3.3.0 of the software available for free download (http://www.cs.princeton.edu/~schapire/maxent/). We accepted recommended default values for convergence threshold (10^−5^), maximum iterations (500) and background points (10,000), since our data set was similar to those used to calculate the tuned settings (i.e. number of environmental variables and number of presence sites) [37]. Similarly, we also used the regularization value (to reduce overfitting) and the combination of feature classes automatically selected by the program [37]. Since we modelled a species expanding its range (i.e. non at equilibrium), the background sample required by Maxent was restricted to that area reachable by the species in order to obtain better predictions [36]. We considered as reachable area that between the early record and the furthest griffon vulture presence data (i.e. roost or breeding colony) towards the west (i.e. the main direction of expansion of the species in the study area during the last two decades; authors, unpubl. data).

To reduce multicolinearity between variables, we calculated the Spearman's correlation coefficients (r_s_). From a pair of variables highly correlated (r_s_>0.5), we selected the variables with a higher ecological significance according to both the biology of the species and the scale considered [54].

### Model evaluation

The importance of using more than one metric to assess model performance, because each quantifies a different aspect of predictive performance, has been previously highlighted [58]. Accordingly, we used seven different metrics to assess model predictions. The area under the Receiver Operating Characteristics curve (AUC) measures the ability of a model to correctly rank a site where the species is present vs. that where it is absent. The AUC varies from 0.5 for models with no discrimination ability to 1 for models with perfect discrimination [59]. It was calculated using the ROCR package [60]. The correlation coefficient (COR) takes into account how far the prediction varies from the observation and can be calculated as a Pearson correlation coefficient [8]. We also used threshold-dependent statistics [53], [61]. Cohen's Kappa provides an index of model accuracy considering omission and commission errors. It ranges from −1 to +1, with +1 indicating maximum accuracy and 0 an accuracy no better than random. Despite being the most widely used measure for the performance of models generating presence–absence predictions, several studies have argued that the kappa statistic introduces statistical artifacts to estimates of predictive accuracy [61]. Accordingly, we also calculated the true skill statistic (TSS), an alternative measure of accuracy which compensates for the shortcomings of kappa while keeping all of its advantages [61]. TSS takes into account both omission and commission errors and ranges from −1 to +1, where +1 indicates perfect agreement and values of zero or less indicate a performance no better than random [61]. We also used the correct classification rate, which indicates the proportion of correctly classified sites (presence/absence) of the test data for a given threshold. Sensitivity and specificity were considered to evaluate how well the model classified presences and absences respectively. To perform these analyses, we must select a threshold to reclassify the model into a binary map (i.e. presence/absence). The selection of an appropriate threshold level above which to consider the species as present is a common concern in presence-only modelling, as no general method for establishing these thresholds has been developed [62]. We selected the 10^th^ percentile presence value because it had the lowest p-value for the null hypothesis that test points are predicted no better than by a random prediction with the same fractional predicted area. Moreover, this threshold corresponded also to the value which maximised the sum of sensitivity (i.e. proportion of observed presences correctly predicted) and specificity (i.e. proportion of observed absences correctly predicted), the criteria producing the most accurate presence/absence predictions [63]. All statistical analyses were performed with R 2.9.2 [64].

Additionally, model performance was also evaluated using the default method of determining the area under the receiver operating characteristic curve (ROC) for the Maxent model. The AUC was calculated for both a training and a test data set, after partitioning the data by randomly assigning 30% of presences to test (i.e. test data set) and the remaining 70% to train the model (i.e. training data set) [6].

Spatial autocorrelation in model residuals (i.e. observed occurrence-probability of occurrence given by Maxent) was investigated by examining Moran's correlogram of residuals, which plots the Moran's Index (*I*) coefficients against distances between localities [65]. This index indicates the degree of similarity/dissimilarity between the values of the residuals in this case. Distance classes for the correlogram were defined maximizing the similarity in the number of interactions between pairs of localities [66]. To test the significance of these Moran's coefficients for each lag distance, 9,999 Monte Carlo permutations of the model residuals were performed and its *P* values were calculated [67]. The Moran's correlogram as a whole is considered significant if at least one of its coefficients is significant at the probability level after progressive Bonferroni correction (here *P*≤0.005). The distance classes, Moran's *I* statistics and correlogram were computed using the freeware package SAM (Spatial Analysis in Macroecology) [68].

## Supporting Information

Figure S1 Spatial correlogram of Moran's I for model residuals. White circles represent non-significant values at the probability level after sequential Bonferroni correction (i.e. P<0.005).
(0.26 MB DOC)
