# Supplementary material for: Anticipating Knowledge to Inform Species Management: Predicting Spatially Explicit Habitat Suitability of a Colonial Vulture Spreading Its Range
Source: PLoS One. 2010 Aug 25;5(8):e12374. doi: 10.1371/journal.pone.0012374 (PMC2928263; doi:10.1371/journal.pone.0012374)
Supplement: Supplementary file 1 [file pone.0012374.s001.doc]

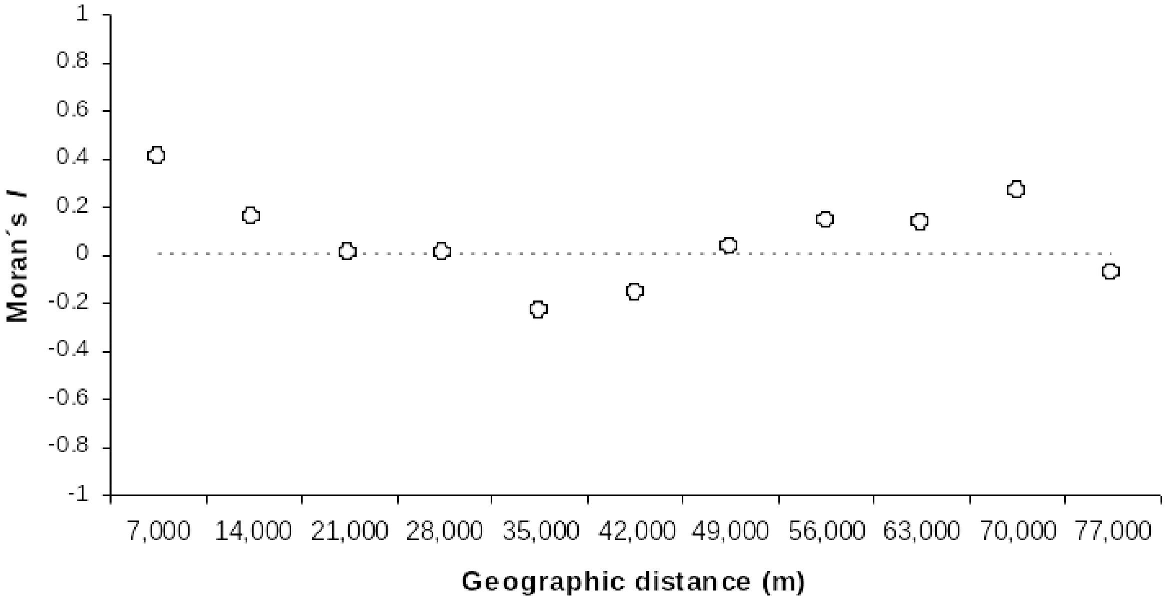

1
0.8
0.6
0.4
0.2
0
-0.2
-0.4
-0.6
-0.8
-1
Moran´s *I*
7,000
14,000
21,000
28,000
35,000
42,000
49,000
56,000
63,000
70,000
77,000
Geographic distance (m)
